# Integrating herbal medicine into mainstream healthcare in Ghana: clients’ acceptability, perceptions and disclosure of use

**DOI:** 10.1186/s12906-017-2025-4

**Published:** 2017-12-01

**Authors:** Peter Agyei-Baffour, Agnes Kudolo, Dan Yedu Quansah, Daniel Boateng

**Affiliations:** 1 School of Public Health, Kwame Nkrumah University of Science and Technology, Kumasi, Ghana; 2 Kumasi Anglican Senior High School, Kumasi, Ghana; 3 Service of Endocrinology, Diabetes and Metabolism, Centre Hospitalier Universitaire Vaudois, University of Lausanne, Lausanne, Switzerland

**Keywords:** Herbal medicine, Integration, Acceptability, Perception, Disclosure, Mainstream healthcare, Kumasi, Ghana

## Abstract

**Background:**

Although there are current efforts to integrate herbal medicine (HM) into mainstream healthcare in Ghana, there is paucity of empirical evidence on the acceptability and concurrent use of HM, in the formal health facilities in Ghana. This study sought to determine client perception, disclosure and acceptability of integrating herbal medicine in mainstream healthcare in Kumasi, Ghana.

**Methods:**

A cross-sectional study was conducted from May to August, 2015. Five hundred patients presenting at the outpatient departments of Kumasi South, Suntreso and Tafo Government Hospitals in Kumasi were randomly selected. Interviews were conducted with the use of structured questionnaires. A logistic regression analysis, using backward selection, was conducted to determine the influence of socio-demographic and facility related factors on the odds of using HM at the facility. All statistical tests were two-sided and considered significant at a *p*-value of <0.05.

**Results:**

Majority of the study respondents were females (64.8%) and the median age was 36 years. Less than half, 42.2%, of the respondents utilized HM services when they visited the health facility. Reasons for using HM at the facility level included ‘being effective’ (24.4%), ‘easy to access’ (25.3%) and ‘being comparatively cheaper’ (16%). About 86% never disclosed previous use of HM to their health care providers. Socio-economic status and perception of service provision influenced use of herbal medicines. Respondents who rated themselves wealthy had increased odds of using herbal medicines at the health facility as compared to those who rated themselves poor (OR = 4.9; 95%CI = 1.6–15.3).

**Conclusion:**

This study shows that integration of herbal medicine is feasible and herbal medicines may be generally accepted as a formal source of healthcare in Ghana. The results of this study might serve as a basis for improvement and upscale of the herbal medicine integration programme in Ghana.

**Electronic supplementary material:**

The online version of this article (10.1186/s12906-017-2025-4) contains supplementary material, which is available to authorized users.

## Background

All over the world, different herbal plants, plant extracts, animal products and mineral substances have been used based on varying cultural backgrounds [1] as a way to manage and treat ill-health, prevent and promote health [2]. Herbal Medicine (HM), also known as Complementary Alternative Medicine (CAM) involves ways of treating and maintaining health that existed before the arrival of orthodox medicine [3]. Anthropologic and cross-cultural perspectives suggests that disease episodes that are recognisable are most likely to be treated outside the perimeter of a formal healthcare system [4].

HM is culturally acceptable and widely utilized in most parts of Africa for a wide spectrum of clinical illnesses [5, 6]. In Ghana, knowledge of HM is almost universal in most homes with evidence of increasing usage, [7] and herbal medicines are used for the treatment and management of both acute (cuts, foot rots) and chronic ailments (stroke, fevers, and diabetes, cancer) [8]. In many parts of the country, herbal medicines are used to either treat malaria or compliment allopathic anti-malaria drugs [9–12]. *Cryptolepis sanguinolenta,* also known as ‘nibima’, have for instance been popularly mentioned and clinically shown to be efficacious against malaria, with its herbal tea formulations, trademarked as Phyto-Laria, being shown to offer 93.5% cure rate in vivo with no signs of toxicity [9, 13]. Research on pregnant women visiting health facilities for anti-natal care also reported use of herbal medicines for the treatment of abdominal pains, constipation, to protect their pregnancies and for smooth delivery [14]. Users of HM are mostly initiated through convincing information from the media, families or friends about the efficacy of previously used herbal medicines [15].

Use of healthcare has been linked to the belief and perceptions about diseases and healthcare [16]. The use of herbs have been associated with common experience, perceptions that herbal medicines were effective, delayed medical care and sufficient knowledge of herbs [14, 17, 18]. Factors such as better efficacy, safe usage, easy access and affordability are also associated with the practice and utilization of herbal medicines over conventional medicines [5, 7, 14, 19, 20]. Other studies have revealed that the use of herbal medicines is independently associated with socio-demographic characteristics such as age, education level, and marital status [17, 18, 21, 22].

In Ghana, efforts have been made to amend the National Health Policy to pave way for the integration of herbal medicine into mainstream healthcare, following the establishment of a policy of herbal medicine practice in 2005 [23]. In 2010, the Traditional Medicine Practice Council (TMPC) was set following the release of the 2nd edition of the Ghana Herbal Pharmacopoeia (GHP) in 2007 [24]. In 2012, HM practice was formally integrated into the main healthcare delivery system in Ghana, with a pilot of about 18 government facilities nationwide [24]. Trained Herbal Medical practitioners from the Kwame Nkrumah University of Science and Technology (KNUST), Centre for Research into Plant Medicine and Tetteh Quarshie Memorial Hospital in Ghana are licensed to consult and prescribe HM for clients both in the government and private hospitals in Ghana. Although the acceptance level of herbal medicines continues to increase, the fact still remains, that there is paucity of data on the acceptability and concurrent application of HM in Ghana. The incidence of combining CAM with allopathic medicines without the knowledge of health professionals may jeopardize therapy as well as cause many side effects or adverse events. This presents important implementation challenges and therefore needs to be assessed. A recent study that assessed the perception of trainers and stakeholders about the integration in one of the pilot facilities [25] revealed a lack of regulatory policy and protocol for integration, leading to different perceptions of the integration and called for multi-facility studies to further look into this. Using quantitative methods and representative samples from three implementing facilities, this study was conducted to determine the acceptability, client perceptions and disclosures related to herbal medicinal use in the public health facilities in the Kumasi metropolis.

## Methods

### Study design and setting

A cross-sectional design was utilized to collect data from May to August 2015. We selected the Kumasi metropolis for this study because the Metropolitan Health Directorate was piloting the concomitant use of herbal and orthodox medicine in some selected health facilities.

The population in Kumasi is heterogeneous as seen by the distinct cultural enclaves with representation of almost all the major ethnic groups in Ghana. The population is dominated by Akans, especially Asantes. According to the 2010 population census report, Kumasi had inter-censual growth rate of 5.4%. The total population of males and females in the metropolis is 972,258 residents and 1,062,806 residents respectively [26]. The metropolis is endowed with 189 health facilities out of which 91% are managed by private individuals. Doctor to patient and nurse to patient ratios are 1:41,606 and 1:7866 respectively and about 81% of the population are registered under the National Health Insurance Scheme (NHIS). Kumasi is the economic nerve of Ghana where people from all over the world converge to do business. Hence findings from studies conducted in Kumasi could be a fair representation of what pertains in Ghana.

### Study population and sampling

The study population included patients who visited outpatient departments in public health facilities (Kumasi South, Suntreso, and Tafo Government hospitals) in Kumasi. Patients who voluntarily agreed and consented were enrolled.

Sample size was estimated using Cochran’s sample size formula [27], ${n}_0=\frac{t{2}^{\ast }(p)(q)}{d2}.$Where t = standard normal deviation = 1.96; p = prevalence rate (assumed to be 50%); q = proportion of target population estimated to access HM from the facility, i.e. 1 – p (50%); and d = degree of accuracy, 5%. This gives a total respondent, n, of 384. Making provision for 10% non-response (1.10 × 384 = 422), and design effect of (1.2 × 422 = 506), the total sample was approximated to 500. The sample drawn from each facility was calculated per their catchment sizes, resulting in 212, 112 and 174 participants from Kumasi South, Tafo and Suntreso Government Hospitals respectively, (See Additional file 1: Table S1).

Study participants were recruited using a systematic random sampling technique, by defining a random starting point and a fixed sampling interval. Based on the number of expected attendance per facility and the period of data collection, we estimated the average respondents needed per day from each facility. On each day, per facility, the sampling interval, *K*, was calculated using the expected attendance and the average respondents needed. During the visit hours, the first participant identified and interviewed as the starting point and the *Kth* respondent is approached, starting the count at the selected starting participant. This was repeated until the required sample size was attained.

### Data collection

Data were collected with the use of structured questionnaires (open ended and closed), which were structured based on the study variables including acceptability, use, and disclosure of use. The study instruments were pretested in a similar public facility, prior to the main data collection, to ascertain participants understanding of the questions before the actual field work started. In circumstances where it became obvious that the respondents found it difficult to answer the questions, the research team re-structured or reframed the questions to enhance clarity. The questionnaires were translated into the local language (Twi) and back translated into English and research assistance were trained to ensure consistency in questioning of participants to enhance uniformity and minimize bias.

Interviews were conducted in serene locations at the Out-Patience Departments (OPDs) of the various health facilities. The interviewers guided the respondents to clearly explain the questions to them where necessary, either in English language or Twi. Participants were assured of confidentiality of their information and were taken through the informed consent processes for approval before being interviewed. All study protocols were reviewed and approved by the Institutional Review Board of the Kwame Nkrumah University of Science and Technology - Committee for Human Research Publications and Ethics (CHRPE) and informed consents was sought from all participants.

### Statistical analysis

All statistical analyses were carried out with Statistical Package for Social Sciences (SPSS) Version 22 [28]. Prior to data entry, any blank fields or inconsistencies in each questionnaire were resolved. General characteristics are summarized as proportions and mean ± standard deviation (SD). Bivariate associations were tested using Pearson and ordinal (linear) Chi-square for categorical and ordinal data respectively. Continuous variables were tested using one way analysis of variance (ANOVA). A logistic regression analysis, using backward selection, was also conducted to determine the influence of socio-demographic and facility related factors on the odds of using herbal medicine at the facility. Three models were fitted; model one tested the influence of socio-demographic variables, model two health facility variables and model 3 a combination of all covariates. All statistical tests were two-sided and were considered significant at a *p*-value of < 0.05.

## Results

### Baseline characteristics of study participants

The median age (25th, 75th percentile) of the subjects was 36 years (28, 49 years) and majority, 64.8% were females, Table 1. Most of the respondents had formal education (senior or middle schools). 56.6% of the respondents were married and 80% had either skilled or semi-skilled jobs. Majority were Christians (85%) and Akans (82.8%).

**Table 1:** Background characteristics of respondents

Variables	Frequency (*N*=500)	Percentage (%)
Age, years		
<25	61	12.2
25-34	156	31.2
35-44	99	19.8
45-54	108	21.6
>54	76	15.2
Median	*36*	
Range	*15-86*	
Sex, Females	324	64.8
Level of education		
None	71	8.2
Basic education (Primary and JSS)	155	14.2
Professional certificate	21	3.0
Senior High School/Middle school	212	4.2
Tertiary	41	42.4
Marital status		
Single	108	21.6
Co-habitation	45	9.0
Married	283	56.6
Divorced/ Widowed	64	12.8
Employment		
Skilled	185	37
Semi-skilled	218	43.6
Unemployed	97	19.4
Religion		
Christian	436	87.2
Muslim	34	6.8
Traditional	8	1.6
None	22	4.4
Ethnicity		
Akan	414	82.8
Ewe	35	7.0
Ga	2	0.4
Others	49	9.8
Self-rated socio-economic status		
Wealthy	71	14.2
Moderately Wealthy	310	62.0
Poor	119	23.8

*SD* Standard deviation; *GHS* Ghana cedi; *JSS* Junior secondary school

### Herbal medicine utilization

About 98.4% of the participants had ever used HM and this was the usual treatment option for 46.2%, Table 2. 42.2% of the respondents utilized herbal medicine when they visited health facilities and 85.8% of them were hoped to utilized this service in the future. Pharmaceutical pre-packaged dosage forms were the commonest (54.2%) source of herbal medicinal products followed by self-prepared formulations (33.8%). Pharmaceutical stores and market places constituted the most popular outlets from which herbal medicinal products were purchased. Majority of the respondents used herbal medicines once a week. The median cost of herbal medicine treatment was GHS 15.00 (USD 3.49) and majority perceived this cost as affordable. The median length of time spent to access herbal medicine in health facilities was 20 min.

**Table 2:** Use of Herbal medicines among study participants

Variables	Frequency *N* = 500	Percentage
Ever used HM	492	98.4
Currently using HM at health facility	211	42.2
Hope to use HM at health facility	429	85.8
Herbal medicine is usual treatment option	231	46.2
Member of your family currently use HM, Yes	210	42.0
Member of your family ever provided care with HM, Yes	358	71.6
Medium of accessing HM apart from health facility		
− Pharmaceutical pre-packaged dosage forms	271	54.2
− Herbalist/spiritual healer formulations	41	8.2
− Self-prepared formulations	169	33.8
− Other	19	3.8
Place HM are purchased (*n* = 492)		
− Hospital	18	3.7
− Pharmaceutical stores	237	49.0
− Supermarket	3	0.6
− Market places	107	22.1
− Itinerant	32	6.6
− Other	87	18.0
Preferred source of HM products		
− Pharmaceutical pre-packaged dosage forms	278	55.6
− Herbalist/spiritual healer formulations	23	4.6
− Self-prepared formulations.	174	34.8
− Other	25	5.0
Frequency of use of HM as treatment option (*n* = 496)		
− Only when sick	279	55.8
− Regularly	79	15.8
− Occasional	112	24.4
− Other	30	6.0
Number of times visited health facility in past 12 months		
− *Median*	2	
− *Range*	0–15	
Number of times sought herbal treatment from facility in last 12 months		
− *Median*	2	
− *Range*	0–9	
Cost of HM usage at the facility, GHC		
− *Median*	15.00	
− *Range*	0.00–500.00	
Perception about cost of seeking HM		
− Affordable	263	52.6
− Not affordable	138	27.6
− Reasonable	99	19.8
Source of payment when use HM to treat your health condition		
− Family members	22	4.4
− Self-financing	363	72.6
− No money required	86	17.2
− Other	29	5.8
Length of time spent to access HM services at the facility (minutes) (*n* = 450)		
− <30	319	63.8
− 30–60	131	26.2
− *Median*	*20.00*	
− *Range*	*0.00–60.00*	

*GHC* Ghana Cedi; *HM* Herbal medicine

### Clients’ impression, satisfaction and reasons for use of integrated herbal medicine services at health facility

**Fig. 1:**
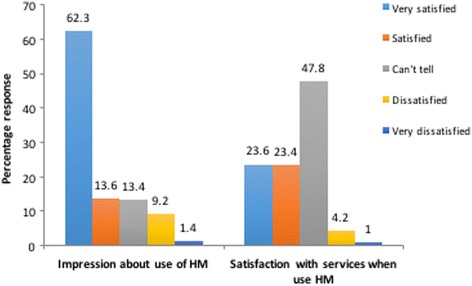
Client’s impression and satisfaction

As shown in Fig. 1, most of the respondents were satisfied with the integrated HM services at the health facility. Majority, 53% of the respondents indicated that preferences for HM would increase if herbal formulations are proven to be an effective treatment option whereas 13% believe positive recommendation of herbal medicines may increase usage. Others believed that people will opt for herbal medicine because of the high cost (11%) and exhaustive access associated with orthodox medicines (5%). 11% also stated that people would prefer to use herbal medicines because of its safety and natural propensity.

### The disclosure between patients and healthcare providers on use of herbal medicine

Most, 98.4% of respondents had never shared information on their use of herbal medicine with orthodox health care providers, although majority (56.9%) believed it was important to do so for treatment efficacy reasons, Table 3. Reasons for non-disclosure of HM use included ‘being unnecessary’ (60.7%) and ‘will be insulted’ (7.8%). About 42.3% were not aware of availability of HM at the health facility, whiles 13.8% and 2.7% obtained information from health professionals and the media respectively. Some respondents disclosed that they struggled to obtain information on the dosage and when to take their medications.

**Table 3:** Disclosures between patients and healthcare providers on use of herbal medicine

Variables	Frequency *N* = 500	Percentage
Ever disclosed or share information on HM used with the health care provider		
− Yes	54	10.8
− No	428	85.6
− Can’t tell	18	3.6
Opinion about the importance of disclosure of HM to health care providers (*n* = 51)		
− Safety	12	23.5
− Efficacy	29	56.9
− Herb-drug interactions	8	15.7
− Other	2	3.9
Reasons for not disclosing use of HM to healthcare provider (*n* = 422)		
− I will be insulted	33	7.8
− It will not be accepted	61	14.5
− It is not necessary	256	60.7
− Other reasons	72	17.1
Source of information about availability of HM at the facility (*n* = 485)		
− Relatives	25	5.2
− Friends	19	3.9
− Health professionals	67	13.8
− Media	13	2.7
− Not aware	205	42.3
− Other	156	32.2
Often struggle to get information on how to take medications when using HM	30	6.0
Perceive relationship with health providers		
− Cordial	215	43.0
− Supportive	229	45.8
− Hostile	11	2.2
− Other	45	9.0

*HM* herbal medicine

### Predictors of herbal medicine usage at the health facility

Employment status, self-rated socio-economic status and satisfaction with services were associated with use of HM at the health facility in the fully adjusted model (Table 4). The odds of using HM at the health facility was higher among respondents who rated themselves as wealthy or as compared to those who rated themselves poor. Respondents who believed the cost of HM were affordable had higher odds of using HM at the facility than those who believed otherwise (OR, 6.7; 95%CI, 2.1, 21.7). The model statistics shows that model 3 (full model) was much improved as compared to 1 and 2.

**Table 4:** Multivariable logistic regression analyses of factors influencing use of  herbal medicine

Covariates	Model 1 AOR [95% CI]	Model 2 AOR [95% CI]	Model 3 AOR [95% CI]
*Socio-demographic factors*			
Employment			
− Unemployed	1		1
− Skilled	0.9 [0.5, 1.7]		0.7 [0.2, 2.4]
− Semi-skilled	0.4 [0.2, 0.7]**		0.1 [0.04, 0.4]**
Religion			
− Christian	1		–
− Muslim	3.1 [1.4, 6.5]**		
− Traditional/ None	1.7 [0.7, 3.9]		
Marital status			
− Single	1		–
− Married/ Co-habitation	0.6 [0.4, 1.0]		
− Divorced/Widow	1.0 [0.5, 2.1]		
Self-rated socio-economic status			
− Poor	1		1
− Wealthy	8.3 [4.0, 17.0]***		4.9 [1.6, 15.3]**
− Moderately wealthy	4.0 [2.2, 7.1]***		10.9 [7.0, 21.9]**
*Health service related factors*			
Satisfaction with the services			
− Dissatisfied/Very dissatisfied		1	1
−		1.3 [0.4, 4.0]	7.3 [2.2, 23.9]**
− Very satisfied		1.1 [1.0, 1.9]*	1.1 [0.4. 2.9]**
− Satisfied			
Perception about cost of seeking HM			
− Not Affordable		1	–
− Affordable/Reasonable		6.7 [2.1, 21.7]**	
Do not have health insurance		1.9 [0.9, 3.5]	2.2 [1.0, 4.9]*
AUC (95% CI)	0.70 (0.66, 0.75)	0.85 (0.81, 0.89)	0.91 (0.88, 0.94)
R^2^	0.18	0.50	0.61
−2 Log likelihood	607.14	405.00	345.77

OUTCOME use of herbal medicine services at the facility; *HM* Herbal medicine; *AOR* Adjusted odds ratio; *p < 0.05; **p < 0.01; ***p < 0.001

## Discussion

This study was conducted to explore the use, perceptions, acceptability and disclosures related to the integrated herbal medicine services in the mainstream healthcare in Ghana. We found a generally high level of herbal medicine usage among respondents. Almost all respondents in this study had ever used herbal medicines and most of these were pharmaceutical pre-packaged forms. Our results corroborates findings of increased use of herbal medicines in most parts of the world [29–31]. A study conducted in the Tanga District in Tanzania, also reported a 42% prevalence of use of herbal medicines [32]. Previous evidence from studies on specific populations have also shown high level of herbal medicine use. For example, study of pregnant women in Nigeria, reported a 67.5% prevalence of herbal medicine usage among pregnant women [33]. In Ghana, pregnant women are reported to use herbal medicines for the treatment of abdominal pains, constipation and to enhance smooth delivery [14]. These results were however dependent on the geographic area surveyed and the socio-cultural characteristics and ethnic background of the surveyed groups.

We further found that, although most patients at the public health facilities have ever used herbal medicines, not all of them were utilizing the integrated herbal medicine services at the health facility level. 42.2% of people, who patronize public health facilities, are currently utilizing the services of herbal medical practitioners at the health facility. This level of acceptability, 3 years into the integration program, however shows positive indications of possible integration of HM into mainstream healthcare. This suggest that, if HM is well integrated into mainstream healthcare, clients could assess both herbal and allopathic treatments in a formal setting for the treatment and management of both acute and chronic diseases. Previous evidence shows success from similar integration programs in other countries [3]. Countries like China and Sri Lanka have successfully integrated these two health systems. In China, 95% of general hospitals offering promotional and curative applications have traditional medicine departments and traditional Chinese medicine is used for the treatment of over 200 million outpatients and almost 3 million inpatients every year [3]. Sri Lanka has also evolved as one of the best health care systems in Asia and has attained health targets almost similar to standards in the Western world as a result of successful healthcare integration [3]. These indicate that with the availability of systematic knowledge, comprehensive methodology and rich clinical experiences, herbal medicines could be used as effective alternatives to complement healthcare provision in Ghana.

### Disclosure of usage of HM

We found that, although most respondents believed that it is important to disclose the use of herbal medicine for efficacy reasons, majority had never done so. Evidence from a systematic review, also confirm a high rate of non-disclosure among users of herbal medicines [34]. Most of the non-disclosers in our study believed it were not necessary to do so whiles others felt they would be insulted by orthodox practitioners for disclosing their use of herbal medicines. In a similar study in the United States, patients reasons for nondisclosure of alternative medicine use included ‘doctors not enquiring of their use of alternative use’ and ‘beliefs that doctors need not know or would not understand’ [35]. A study by Braun et al. [36], also reported ‘concerns of undesirable response by the practitioners’, ‘the perception that the practitioner need not know about their alternative medicine use’, ‘and practitioners not asking about it’ as major reasons for patients lack of disclosure of herbal medicines. It is also believed that perceived legitimacy of alternative medicine treatments affects disclosure [37].

### Client’s satisfaction and use of HM at the health facility

This study also found that satisfaction with services provided influenced the use of HM at the health facilities. Patient satisfaction has been suggested as a major quality outcome [38, 39] and the extent to which they are satisfied with their health providers may be an important consideration in their health behaviour and health care utilization [40, 41]. Our study found a high level of satisfaction with services provided to herbal medicines users at the health facilities. Majority of study subjects were impressed with the service provision of Herbal Medical practitioners.

Most of them also believed herbal medical services were affordable, although this could stem from a general perception in the Ghanaian community, that, herbal medicine is less expensive than orthodox medicines. In rural communities, herbal practitioners are even willing to accept delayed payment, payment in kind such as fowls, agricultural seeds, goats, palm oil, salt, or palm wine, or in some cases patients can negotiate the amount [42]. Buor [43] also argued that herbal medicines are relatively cheaper than modern medicines. This assumption could however hold at the community level, but at the health facility level, with the advent of NHIS, subscribers of the NHIS scheme will see the services of herbal medical practitioners as expensive. The current services at the health facilities are not covered by health insurance. More than 75% paid for the cost of HM by self-financing and the median cost of HM treatment at the facility among users in this study was GHS 15.00 (USD 3.49). It could therefore be postulated that the patronage of HM could increase if the services are covered by the health insurance scheme. As observed in this study, inactive NHIS members were more likely to utilize HM at the health facility as compared to active NHIS members and therefore the extent of utilization could improve if the services are covered by the NHIS [15].

### Predictors of use of herbal medicines at the health facility

This study found that respondents who were semi-skilled had decreased odds of utilizing HM services as compared to those who were unemployed. The study also observed increased propensity of usage of HM among participants who rated themselves wealthy as compared to those who rated themselves poor. Employment and socio-economic status were also significantly associated with utilization of HM in the bivariate analysis (See Additional file 1: Table S2). Evidence from some national surveys have also shown an association between higher socio-economic status and use of CAM, although this was not universal across all racial/ethnic groups [44]. The use of the integrated HM services among high socio-economic class in this setting, could however be due to their ability to pay for their services, which are not covered by the NHIS.

### Strength and limitations

This study provides important quantitative data on the acceptability, use and perceptions of clients on the integrated herbal medicines services in public health facilities in Ghana. The availability of such finding is important to inform and guide the scale up of the programme. The use of participants from three facilities in the pilot programme enhances the generalizability of the study findings and strengthen the evidence for policy advice. Dwelling on previous experiences, this study might suffer some recall bias. We however ensured that appropriate questions were used to tease out responses, thereby ensuring that this bias is reduced to almost negligible. Also, important information on the point or state of disease condition (mild, serious or worse) where patients consider leaving herbal treatment was not assessed in this study. We recommend further studies to look at the point or state of diseases and the consideration of HM as well as expanding the study to cover other regions to gain broader insight into the subject matter.

## Conclusion

We found that 42.2% of the study participants who patronized public health facilities, sort for the services of herbal medical practitioners at the health facility. Three years into the integration programme, this evidence shows a promising level of acceptability of use of HM at the health facility. However, it is an undeniable fact that there are gaps in awareness and reluctance in disclosure to allopathic health providers. Majority of the respondents did not disclose the use of herbal medicines. Whiles this study points towards a possible integration of herbal medicines with allopathic care, there is the need for general education and orientation of health service providers and clients on the availability of HM service in public health facilities. Findings from this study also suggest the need for further education of health providers on the legitimacy and acceptability of herbal medicine to run concurrently with orthodox medicine.

## Additional file

Additional file 1: Table S1. Estimated sample from each facility. **Table S2.** Bivariate analysis of Socio-demographic and healthcare related factors associated with herbal medicine use. (DOCX 74 kb)

## Abbreviations

CAM: Complementary Alternative Medicine
FDA: Foods and Drug Authority
GHP: Ghana Herbal Pharmacopoeia
GHS: Ghana Health Service
GSA: Ghana Standard Authority
HM: Herbal Medicine
KNUST: Kwame Nkrumah University of Science and Technology
MOH: Ministry of Health
NHIA: National Health Insurance Scheme
TMPC: Traditional Medicine Practice Council
WHO: World Health Organization
