# Supplementary material for: Integrating herbal medicine into mainstream healthcare in Ghana: clients’ acceptability, perceptions and disclosure of use
Source: BMC Complement Altern Med. 2017 Dec 1;17:513. doi: 10.1186/s12906-017-2025-4 (PMC5709853; doi:10.1186/s12906-017-2025-4)
Supplement: Additional file 1: Table S1. — Estimated sample from each facility. Table S2. Bivariate analysis of Socio-demographic and healthcare related factors associated with herbal medicine use. (DOCX 74 kb) [file 12906_2017_2025_MOESM1_ESM.docx]

**Supplementary Table 1:** Estimated sample from each facility.

| **Hospital** | **Catchments size** | **Proportion (%)** | **Sample** |
| --- | --- | --- | --- |
| Kumasi South | 448, 097 | 42.97 | 215 |
| Tafo hospital | 236, 619 | 22.69 | 113 |
| Suntreso Government Hospital | 357, 886 | 34.33 | 172 |
| **Total** | **1,042,602** | **100** | **500** |

**Supplementary Table 2.** Socio-demographic and healthcare related factors associated with herbal medicine use

| **Variables** | **Use Herbal Medicine at facility** | | **Chi-square/F-statistic** | **p-value** |
| --- | --- | --- | --- | --- |
|  | **Yes, %**  **(n=211)** | **No, %**  **(n=289)** |  |  |
| **Age**   - <25 - 25-34 - 35-44 - 45-54 - >54 | 55.7  37.8  44.4  41.7  38.2 | 44.3  62.2  55.6  58.3  61.8 | 6.536 | 0.163 |
| **Gender**   - Female - Male | 39.2  47.7 | 60.8  52.3 | 3.402 | 0.065 |
| **Level of education**   - None - Basic education (Primary and JSS) - Senior High School/Middle school - Professional certificate /Tertiary | 36.6  41.3  40.6  56.5 | 63.4  58.7  59.4  43.5 | 6.345 | 0.096 |
| **Marital status**   - Single - Married/ Co-habitation - Divorced/Widow | 54.6  39.0  37.5 | 45.4  61.0  62.5 | 8.776 | 0.012 |
| **Employment**   - Skilled - Semi-skilled - Unemployed | 55.1  30.3  44.3 | 44.9  69.7  55.7 | 25.580 | <0.001 |
| **Religion**   - Christian - Muslim - Traditional/ None | 40.8  55.9  46.7 | 59.2  44.1  53.3 | 3.193 | 0.203 |
| **Self-rated socio-economic status**   - Wealthy - Moderately wealthy - Poor | 66.2  43.9  23.5 | 33.8  56.1  76.5 | 34.124 | <0.001^¥^ |
| **Monthly income, mean (SD)** | 479.90 | 531.89 | 1.359 | 0.244^§^ |
| **Ever used Herbal Medicine**   - Yes - No | 42.1  50.0 | 57.9  50.0 | 0.203 | 0.652 |
| **Perception about cost of seeking HM**   - Affordable - Not affordable - Reasonable | 33.8  47.1  62.0 | 66.2  52.9  38.0 | 20.295 | <0.001^¥^ |
| **NHIS status**   - Active - Inactive | 39.4  49.0 | 60.6  51.0 | 3.924 | 0.048 |
| **Member of your family ever provided care with HM**   - Yes - No | 48.3  27.1 | 51.7  72.9 | 18.492 | <0.001 |

*^¥^Estimated by ordinal (linear) Chi-square; ^§^Estimated by one way ANOVA*
